# Intra- and inter-operator reproducibility of US point shear-wave elastography in various organs: evaluation in phantoms and healthy volunteers

**DOI:** 10.1007/s00330-019-06195-8

**Published:** 2019-05-14

**Authors:** Riwa Kishimoto, Katsuhiko Kikuchi, Atsuhisa Koyama, Jeff Kershaw, Tokuhiko Omatsu, Yasuhiko Tachibana, Mikio Suga, Takayuki Obata

**Affiliations:** 1 National Institute of Radiological Sciences, National Institutes for Quantum and Radiological Science and Technology, 4-9-1 Anagawa, Chiba, 263-8555 Japan; 2 Tokyo-Kita Medical Center, 4-17-56, Akabanedai, Tokyo, 115-0053 Japan; 3 Center for Frontier Medical Engineering, Chiba University, 1-33, Yayoi-chou, Chiba, 263-8522 Japan

**Keywords:** Elasticity imaging techniques, Observer variation, Reproducibility of results, Ultrasonography

## Abstract

**Purpose:**

This study was conducted in order to assess the intra- and interoperator reproducibility of shear-wave speed (SWS) measurement on elasticity phantoms and healthy volunteers using ultrasound-based point shear-wave elastography.

**Materials and methods:**

This study was approved by the institutional review board. Two operators measured the SWS of five elasticity phantoms and seven organs (thyroid, lymph node, muscle, spleen, kidney, pancreas, and liver) of 30 healthy volunteers with 1.0–4.5 MHz convex (4C1) and 4.0–9.0 MHz linear (9L4) transducers. The phantom measurements were repeated ten times, while the volunteer measurements were performed five times each. Intra- and interoperator reproducibility was assessed. Interoperator reproducibility was also evaluated with the 95% Bland–Altman limits of agreement (LOA).

**Results:**

In phantoms, all intraclass correlation coefficients (ICCs) were above 0.90 and the 95% LOA between the two operators were less than ± 18%. In volunteers, intraoperator ICCs were > 0.75 for all regions except the pancreas. Interoperator ICC was above 0.75 for the right lobe of the liver (depth 4 cm) and the kidney, but the 95% LOA was less than ± 25% only for the liver.

**Conclusion:**

Although excellent in phantoms, interoperator reproducibility was insufficient for all regions in the volunteers other than the right hepatic lobe at a depth of 4 cm. Clinicians should be aware of the 95% LOA when using SWS in patients.

**Key Points:**

*• Our phantom study indicated a high reproducibility for shear-wave speed (SWS) measurements with point shear-wave elastography (pSWE).*

*• In volunteers, intraoperator reproducibility was generally high, but the interoperator reproducibility was not high enough except for the right hepatic lobe at 4 cm depth.*

*• To evaluate interoperator reproducibility, the 95% limits of agreement (LOA) between operators should be considered in addition to the intraclass correlation coefficient (ICC).*

**Electronic supplementary material:**

The online version of this article (10.1007/s00330-019-06195-8) contains supplementary material, which is available to authorized users.

## Introduction

Ultrasound (US) elastography has been widely used to evaluate liver fibrosis or to differentiate between benign and malignant lesions in breast, thyroid, and prostate [1–4]. Measurements of shear-wave speed (SWS) provide quantitative information about organ stiffness, which is a potential noninvasive biomarker [5]. The Quantitative Imaging Biomarkers Alliance (QIBA) organized by the Radiological Society of North America has selected SWS as a potential biomarker. Currently, efforts are underway to make a profile or an implementation guide to achieve sufficient accuracy and avoid variability in the measurement [6].

There are three methods for SWS measurement: (i) transient elastography (TE), which is 1D elastography without an anatomic B-mode image guide [7]; (ii) point shear-wave elastography (pSWE), which provides single-point measurement within a B-mode image; and (iii) 2D color-coded shear-wave elastography (2D SWE), which produces 2D color-velocity maps and allows for multiple measurements to be obtained. pSWE and 2D SWE apply the acoustic radiation force impulse (ARFI) technique, which is based on the emission of short-duration acoustic pulses into tissues that induce localized tissue displacement, resulting in shear-wave propagation away from the region of excitation [8–10].

While TE has been standardized and validated in numerous centers worldwide, its application is limited to organs directly beneath the transducer, and it is seldom used outside the liver, spleen, and kidney. On the other hand, although the main clinical applications of pSWE and 2D SWE are to evaluate liver fibrosis [11–13] and breast [14] and thyroid nodules [15], they can also be applied for the evaluation of inflammatory and neoplastic diseases in the pancreas [16], kidney [17, 18], prostate [19], lymph node [20], and muscles [21]. Many studies have evaluated the accuracy of SWS measurement as a biomarker for staging liver fibrosis or differentiating a malignant lesion from a benign one [8, 9, 11, 14–16, 18, 20]. SWS is also used as a noninvasive biomarker for assessing the therapeutic response of antifibrotic, antiviral, or anticancer drugs [22, 23]. Before SWS can be reliably used as a biomarker for clinical diagnosis, it is essential to estimate the reproducibility of measurements. To date, however, other than for the measurement of liver stiffness [24], the number of studies that specifically address the intra- and interoperator reproducibilities of SWE is limited [25, 26].

The aim of this study is to examine the intra- and interoperator reproducibility of SWS measurement with pSWE in elasticity phantoms and the organs of healthy volunteers.

## Materials and methods

### Subjects

#### Phantoms

Five rectangular parallelepiped phantoms (9 × 13 × 13 cm^3^), suitable for MR and US elastography, were made. These were acrylamide-based homogeneous phantoms containing graphite particles [27]. The storage moduli were measured with a rheometer, and as SWS is proportional to the square root of the stiffness, the corresponding SWSs of the phantoms were also calculated (Table 1). Phantom homogeneity was confirmed with MR elastography, and the corresponding SWSs were also estimated (Table 1) [27].

**Table 1:** Storage modulus and shear-wave speed of five phantoms measured with a rheometer and MR elastography

Storage modulus (kPa)	2.1	5.2	9.7	13.3	25.0
SWS (m/s)	1.41	2.23	3.01	3.56	4.86
SWS w/ MRE (m/s)	1.72 ±0.05	2.42 ±0.01	3.31 ± 0.02	3.75 ± 0.05	4.03 ± 0.04

*SWS* = shear-wave speed, *MRE* = MR elastography

#### Healthy volunteers

This study was approved by the institutional review board and written informed consent was obtained from all participants.

Thirty healthy volunteers (female:male = 15:15, mean age 38.5 years, range 21–58 years; mean body mass index (BMI), 21.7 kg/m^2^, range 16.7–29.5 kg/m^2^) without a history of liver, pancreatic, renal, or thyroid disease were included in this study. The volunteers were asked to fast for at least 4 h prior to examination.

### Imaging techniques

An Acuson S2000 US system (Siemens Healthineers) with 1.0–4.5 MHz convex (4C1) and 4.0–9.0 MHz linear (9L4) transducers was used. SWS measurements were obtained with the virtual touch quantification (VTQ) software, which is Siemens’ implementation of the pSWE method, utilizing the ARFI technique. The region of interest (ROI) was 10 × 6 mm^2^ for the 4C1 transducer and 5 × 5 mm^2^ for the 9L4 transducer. The room temperature was maintained at 20 ^°^C.

Examinations were performed by two operators in separate sessions. Each observer made the required number of measurements for a phantom or organ in successive measurements over a period of 1 or 2 min with minimum movement of the transducer, then moved on to the next phantom or organ. For the phantom studies, one operator was a board-certified radiologist and the other a graduate student of medical engineering. Each operator measured the SWS in a ROI of the same area at four different depths (2, 4, 6, and 8 cm with the 4C1 transducer and 1, 2, 3, and 4 cm with the 9L4 transducer) for each phantom. The measurements were repeated ten times at each depth. Because the hardest phantom was outside of the measurable range of the 4C1 transducer, it was measured only with the 9L4 transducer. Occasionally, SWS measurement was unreliable or out of range (i.e., SWS is displayed as “x.xx m/s” on this system), which was taken to indicate measurement failure. Attempts were made to obtain ten valid measurements, but when the failure rate, which was calculated as the number of measurement failures divided by the number of measurements, was more than 50%, the data was excluded from further evaluation.

**Fig. 1:**
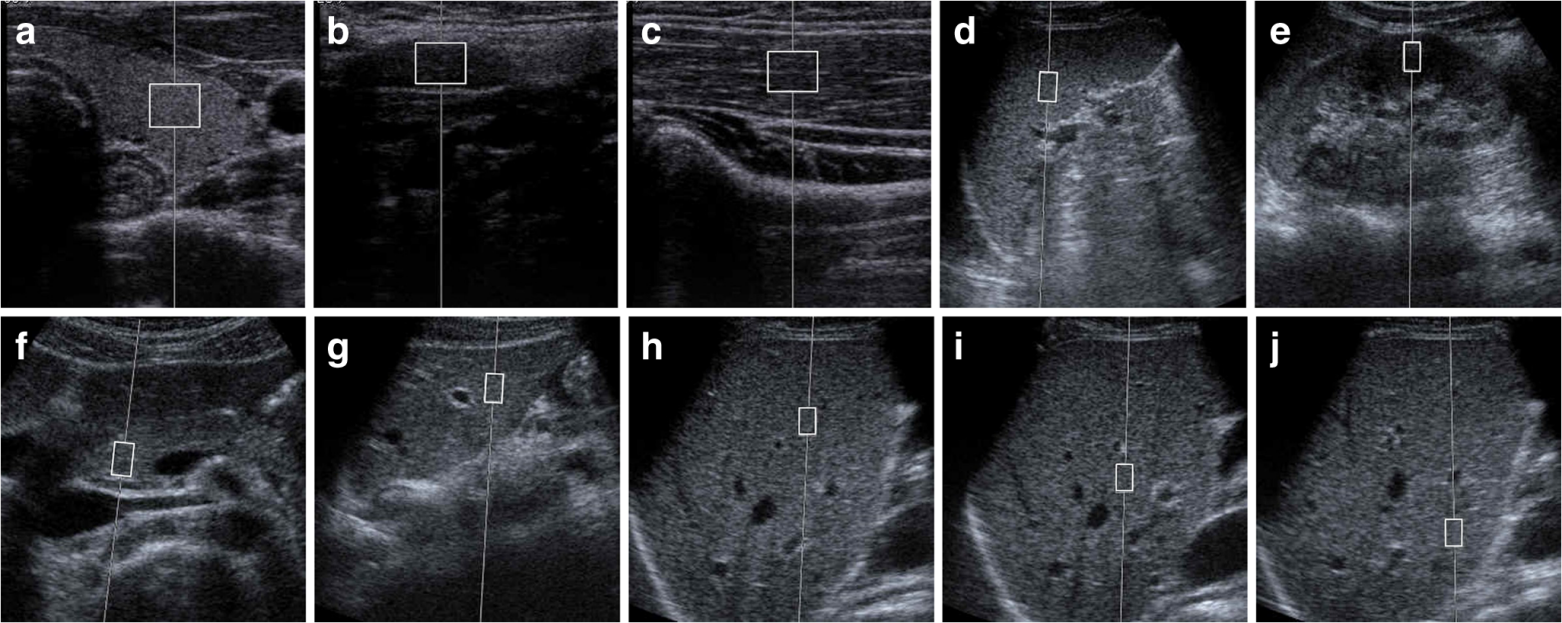
ROI placement for SWS measurement in ten regions of seven organs: (**a**) thyroid; (**b**) cervical lymph node; (**c**) brachioradialis muscle; (**d**) spleen; (**e**) left kidney; (**f**) pancreas; (**g**) left lobe of liver; (**h**) right lobe of liver, 4 cm; (**i**) right lobe of liver, 6 cm; (**j**) right lobe of liver, 8 cm

For the volunteer studies, a board-certified radiologist and a registered medical sonographer measured SWS. Both had more than 20 years of experience in ultrasound examination. The operators measured the SWSs for seven organs: left lobe of the thyroid, cervical lymph node, right brachioradialis muscle, spleen, left kidney, pancreas, and liver (Fig. 1). For the liver, SWS measurements were obtained in the right lobe at three different depths (4, 6, and 8 cm), while only one measurement was made in the left lobe. In accordance with the results of the phantom study and the minimum measurement number described in the guidelines of the World Federation for Ultrasound in Medicine and Biology [1], measurements were repeated five times in each region for the volunteer study. Thus, a total of 100 SWSs (2 operators × 10 regions × 5 times) were obtained for each volunteer. Examination was performed with the 9L4 transducer for the thyroid, lymph node, and brachioradialis muscle, and the 4C1 transducer was used for the spleen, left kidney, pancreas, and liver. The volunteers were placed in a supine position and the SWSs of the abdominal organs were measured during breath-holding after subtle inspiration. The spleen, left kidney, and right hepatic lobe were scanned with an intercostal approach, while the pancreas and left hepatic lobe were scanned with a subcostal approach. Care was taken not to include any blood vessels or biliary structures. For the kidney, the operators were asked to put the ROI in the renal cortex to avoid the medulla. SWSs were measured parallel to the long axis for the lymph node and muscle and perpendicular for the thyroid (Fig. 1).

### Statistical analysis

For the volunteer study, the within-population interquartile range (IQR) was estimated to assess the heterogeneity of the population.

To evaluate intraoperator reproducibility, the one-way random, absolute agreement, average-measure ICC (intraclass correlation coefficient) was calculated using both five and ten measurements for the phantom study (ICC (1, 5) or (1, 10)), and using all five measurements for the volunteer study (ICC (1, 5)). To assess interoperator reproducibility, the two-way random, absolute agreement, average-measure ICC was calculated using the mean SWS produced by each operator (ICC (2, 2)). Measurement reliability was classified according to common criteria as excellent (ICC > 0.75), good (ICC = 0.60–0.75), fair (ICC = 0.40–0.59), and poor (ICC ≤ 0.40) [28].

A Bland–Altman (BA) plot was also used to demonstrate operator-related variations. To aid comparison across regions, the normalized difference, expressed as the percentage difference between the measurements made by each operator, i.e., %ΔSWS = (SWS_1_ − SWS_2_) / (SWS_1_ + SWS_2_) × 100, was calculated, where SWS_*i*_ is the mean measurement made by operator *i*. The 95% limits of agreement (LOA) between the two operators were also calculated as 1.96× the standard deviation of all measurements.

Statistical analyses were carried out using version 24.0 of the SPSS software package (SPSS).

## Results

### Data acquisition

In the phantom study, measurement error occurred at a depth of 1 cm with the 9L4 transducer and at 2 cm with the 4C1 transducer, with the failure rates being 60–100% (mean 85%) and 55–70% (mean 62.5%), respectively. This data was abandoned. Therefore, a total of 15 data sets (at depths of 2, 3, and 4 cm in five phantoms) with the 9L4 transducer and 12 data sets (4, 6, and 8 cm in four phantoms) with the 4C1 transducer were included for analysis.

In the volunteer study, the SWSs of the thyroid, brachioradialis muscle, spleen, left kidney, pancreas, the left hepatic lobe, and right hepatic lobe at depths of 4 and 6 cm were measured successfully for all 30 volunteers. One volunteer had no cervical lymph node of sufficient size for SWS measurement. The long- and short-axis diameters of the other 29 cases were in the ranges 10.8–28.4 mm (mean 15.5 mm) and 4.0–9.4 mm (mean 5.4 mm), respectively. The failure rates for each region are shown in Table 2. Also, valid SWSs at 8 cm depth in the right hepatic lobe were not obtained for one volunteer. The failure rates for this patient were 100 and 50% for the two operators at a depth of 8 cm, while the failure rates for the other 29 volunteers were in the range 0–28.6% (mean 0.8%). Therefore, SWS in the lymph node and at a depth of 8 cm in the right hepatic lobe was evaluated for only 29 subjects. The measurement depth in the left lobe was in the range 2.1–6.6 cm (mean 4.0 cm).

### SWS of phantoms and healthy volunteers

**Fig. 2:**
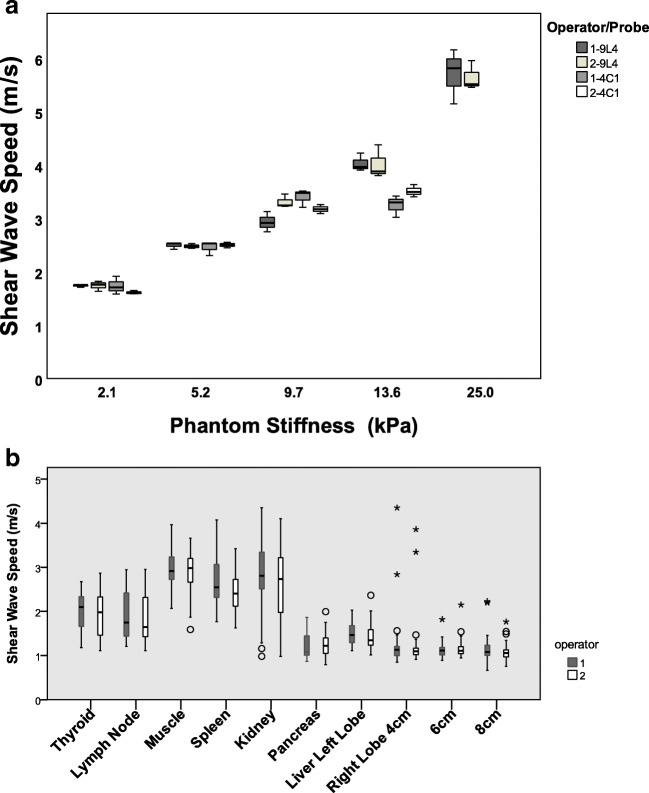
Box and whisker plots of the shear-wave speed for five phantoms (**a**) and across volunteers (**b**) measured by two operators using two different transducers. The phantom stiffness was measured by rheometer. Open circle = outliers, asterisk = extremes

The median and IQR of the SWSs for the five phantoms and volunteers are summarized in Fig. 2 and Tables 2 and 3. Note that the IQR of the phantoms demonstrates the variation of measurements in the same phantom, while the IQR for the volunteers corresponds to the variation across subjects. In addition to the cases where valid SWSs were not obtained (i.e., the measurement failure rate was too high), the extreme values found in the right hepatic lobe (corresponding to more than three times the IQR) were found for two volunteers at 4 cm depth, one volunteer at 6 cm depth, and a further three volunteers at 8 cm depth. All of these data were excluded from further analysis. The BMIs of the volunteers for whom either measurement failure or extreme values were observed were 25.5, 26.0, 27.2, and 29.5. Consequently, the ICC and BA plot were evaluated for only 28, 29, and 26 volunteers for the 4-, 6-, and 8-cm ROIs, respectively, in the right hepatic lobe. No extreme values were found for the other organs.

**Table 2:** The number of cases, failure rate, median, and IQR of the SWS across volunteers for ten regions in seven organs

	No. of cases	Failure rate (%)	Median (m/s)		IQR (m/s)	
			Operator 1	Operator 2	Operator 1	Operator 2
Thyroid	30	0.3 [0–16.7]	2.12	1.98	0.65	0.84
Lymph node	29	0 [0–0]	1.82	1.65	0.96	0.86
Muscle	30	0.3 [0–16.7]	2.91	2.97	0.51	0.54
Spleen	30	1.0 [0–28.6]	2.51	2.39	0.75	0.59
Kidney	30	7 [0–37.5]	2.81	2.73	0.82	1.20
Pancreas	30	4.2 [0–37.5]	1.08	1.21	0.43	0.35
Liver Lt lobe	30	0.3 [0–16.7]	1.45	1.36	0.39	0.34
Liver Rt lobe 4 cm	30	0.8 [0–50.0]	1.13	1.10	0.2	0.14
Liver Rt lobe 6 cm	30	1.4 [0–37.5]	1.11	1.11	0.17	0.15
Liver Rt lobe 8 cm	29	0.8 [0–28.6]	1.08	1.06	0.23	0.16

Failure rate is presented as mean [min–max]

*IQR* = interquartile range, *SWS* = shear-wave speed

**Table 3:** Median and IQR for the SWS of five phantoms

	Probe	Median (m/s)		IQR (m/s)	
		Operator 1	Operator 2	Operator 1	Operator 2
2.1 kPa	9L4	1.76	1.76	0.02	0.09
	4C1	1.71	1.60	0.17	0.03
5.2 kPa	9L4	2.53	2.47	0.06	0.05
	4C1	2.53	2.51	0.11	0.05
9.7 kPa	9L4	2.92	3.25	0.19	0.11
	4C1	3.48	3.17	0.15	0.08
13.3 kPa	9L4	3.97	3.77	0.16	0.29
	4C1	3.30	3.50	0.20	0.11
25.0 kPa	9L4	5.82	5.51	0.51	0.25

### Reproducibility of SWS measurement

**Fig. 3:**
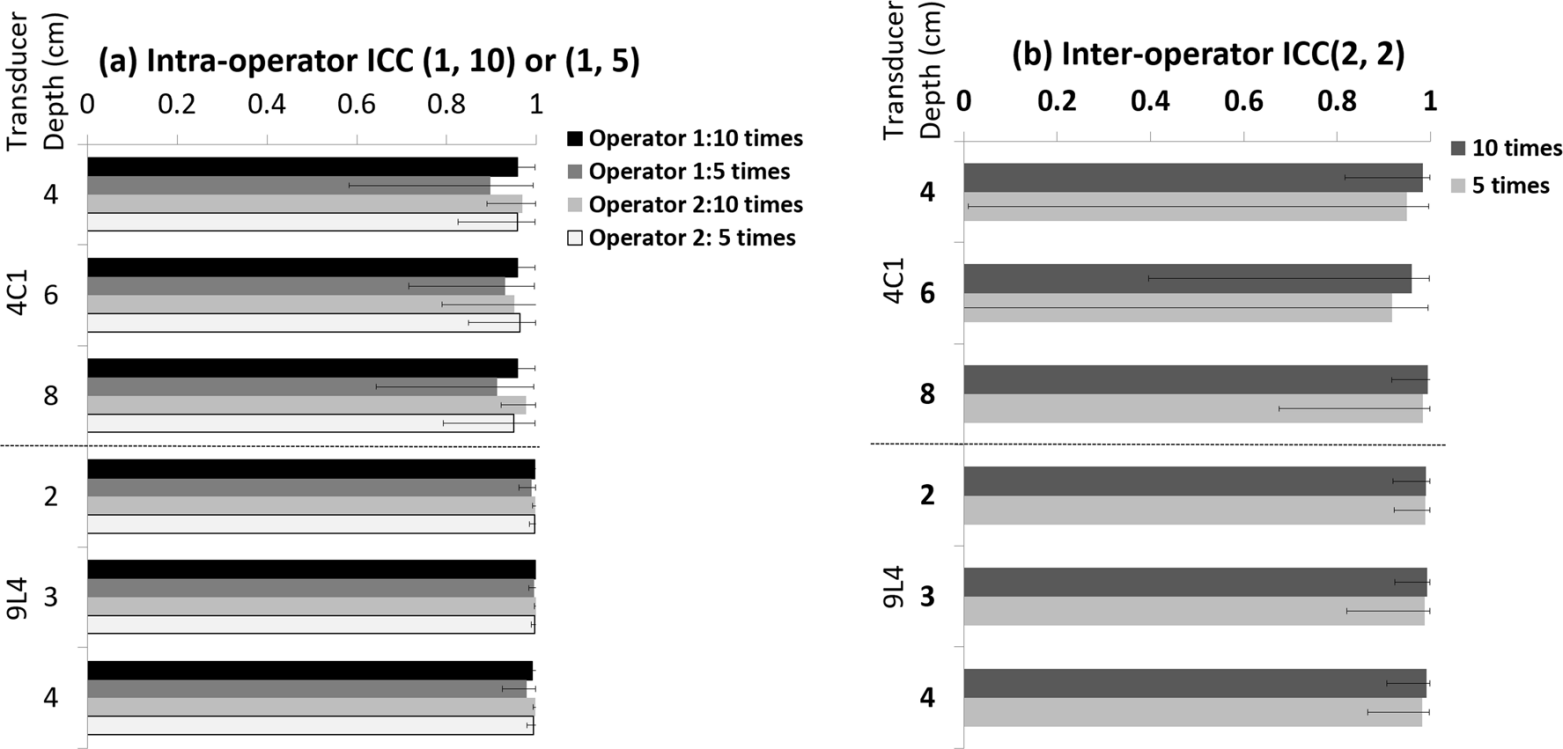
Intra- and interoperator intraclass correlation coefficient (ICC) for the phantom studies. For intraoperator reproducibility (**a**), the one-way random, absolute agreement, average-measure ICC (1, 10) or ICC (1, 5) was calculated, and for interoperator reproducibility **b**, the two-way random, absolute agreement, average-measure ICC (2, 2) was calculated

**Fig. 4:**
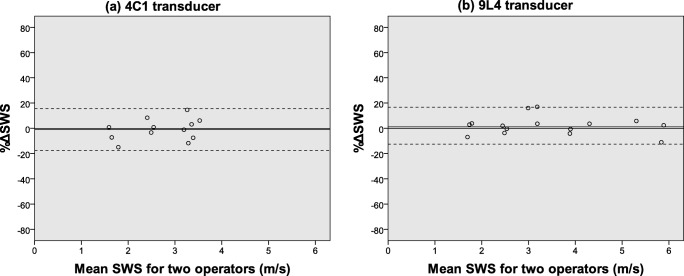
Bland–Altman plot for the interoperator reproducibility of SWS measurement in phantoms with a 4C1 transducer (**a**) and a 9L4 transducer (**b**). The *X*-axis corresponds to the average SWS for two operators and the *Y*-axis is %ΔSWS. SWS = shear-wave speed, solid line = mean bias, dashed line = 95% limits of agreement (1.96× standard deviation)

The intra- and interoperator ICCs for the phantom study using either five or ten measurements are summarized in Fig. 3. All ICCs were larger than 0.90, but ICCs calculated with ten measurements were generally higher than those obtained with only five measurements. A BA plot showing the %ΔSWS and 95% LOA between the two operators calculated from ten measurements is presented in Fig. 4. The 95% LOA were −  17.7~15.7% for the 4C1 transducer and − 12.6~16.7% for the 9L4 transducer.

**Fig. 5:**
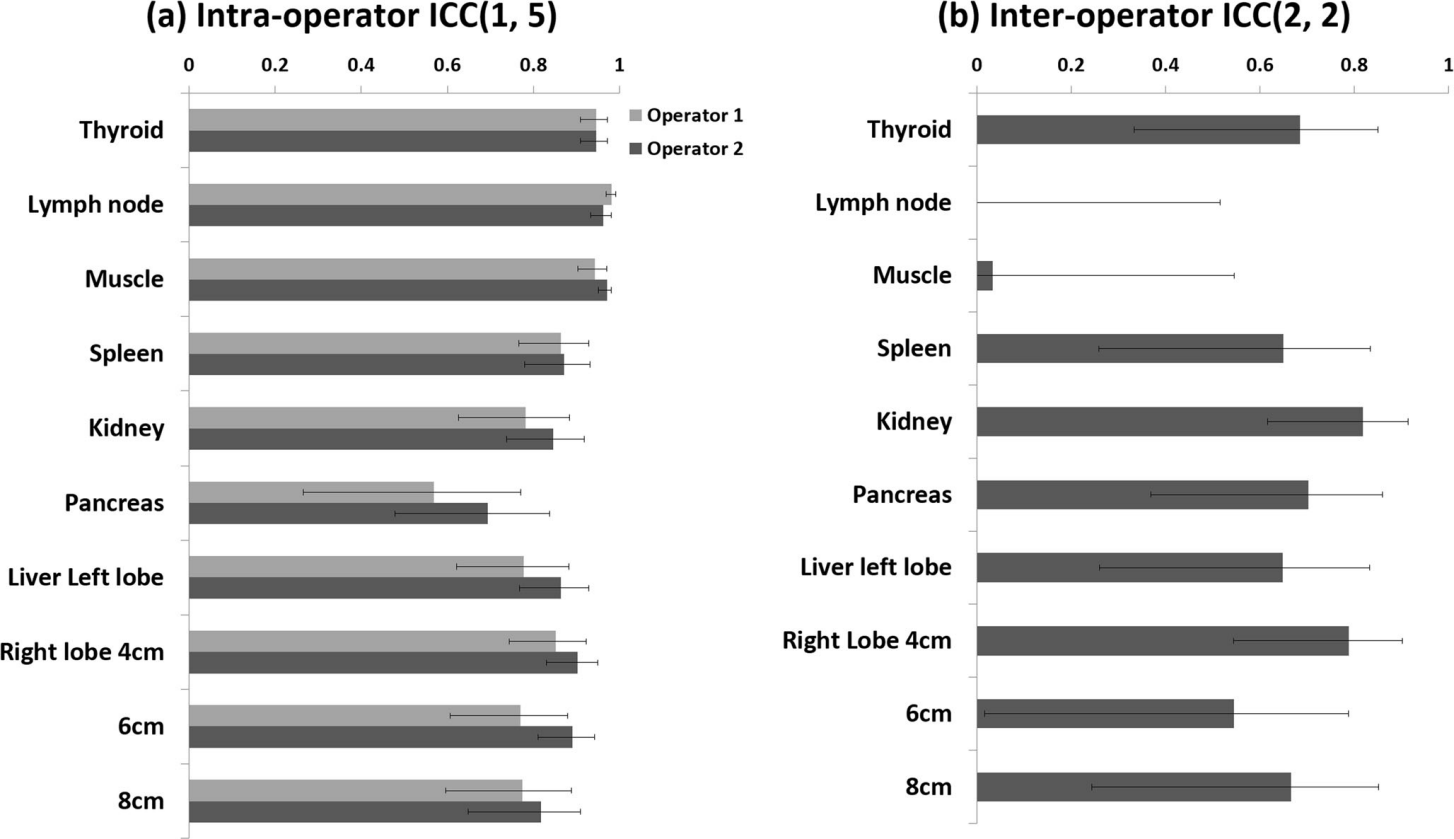
Intra- and interoperator intraclass correlation coefficient (ICC) for ten regions in seven organs. For intraoperator reproducibility (**a**), the one-way random, absolute agreement, average-measure ICC (1, 5) was calculated, and for interoperator reproducibility (**b**), the two-way random, absolute agreement, average-measure ICC (2, 2) was calculated

**Fig. 6:**
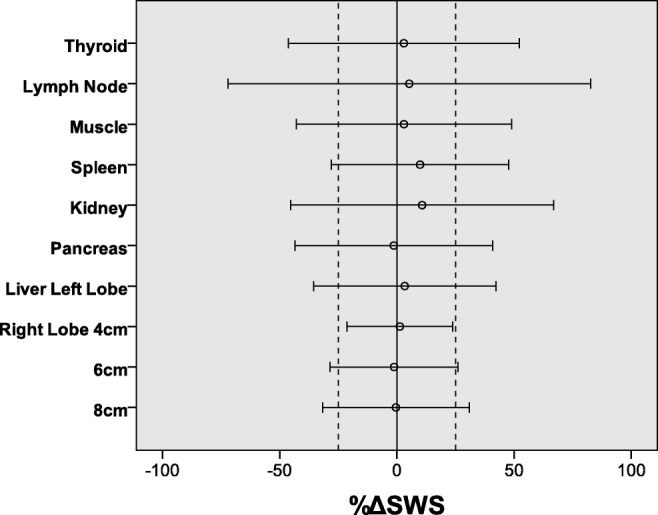
The %ΔSWS between two operators relative to their mean and 95% limits of agreement. SWS = shear-wave speed, dashed line = 25%. %ΔSWS = (SWS_1_ − SWS_2_) / (SWS_1_ + SWS_2_) × 100, where SWS_*i*_ is the mean measurement made by operator i

The intra- and interoperator reproducibilities for ten regions in seven organs are summarized in Fig. 5 and Table 4. The ICCs for intraoperator reproducibility were excellent (i.e., more than 0.75) for all regions except the pancreas, which had ICCs of 0.57 and 0.70 indicating fair to good. For the interoperator reproducibility, an excellent ICC was found only in the kidney and at a 4-cm depth in the right hepatic lobe, while values lower than 0.4 were found for the cervical lymph node and brachioradialis muscle. A BA plot for each of the ten regions is presented in the Supplemental figure, and the %ΔSWS and 95% LOA between the two operators are summarized in Fig. 6. The 95% LOA for the right hepatic lobe at a depth of 4 cm was the only one less than 25%. On the other hand, the 95% LOA was − 43.5~66.9% for the kidney even though the interoperator ICC was excellent (Table 4).

**Table 4:** Intra- and interoperator reproducibilities across volunteers for ten regions in seven organs

	MHz	Intraoperator ICC		Interoperator reproducibility		
		Operator 1	Operator 2	ICC	Mean bias (%)	95% LOA (%)
Thyroid	9	0.95 [0.91–0.97]	0.95 [0.91–0.97]	0.69 [0.33–0.85]	2.96	− 46.0~52.2
Lymph node	9	0.98 [0.97–0.99]	0.96 [0.99–0.98]	0 [0–0.52]	5.27	− 72.1~82.6
Muscle	9	.94[.90–.97]	0.97 [0.95–0.98]	0 [0–0.55]	3	− 42.9~48.9
Spleen	4	0.86 [0.77–0.93]	0.87 [0.78–0.93]	0.65 [0.26–0.83]	9.87	− 28.0~47.7
Kidney	4	0.78 [0.63–0.88]	0.85 [0.74–0.92]	0.82 [0.62–0.91]	10.8	− 45.3~66.9
Pancreas	4	0.57 [0.27–0.77]	0.69 [0.48–0.84]	0.70 [0.37–0.86]	− 1.35	− 43.8~40.8
Liver Lt lobe	4	0.78 [0.62–0.88]	0.86 [0.77–0.93]	0.65 [0.26–0.83]	3.34	− 35.6~42.2
Liver Rt lobe 4 cm	4	0.85 [0.74–0.92]	0.90 [0.83–0.95]	0.79 [0.54–0.90]	1.26	− 21.3~23.8
Liver Rt lobe 6 cm	4	0.77 [0.61–0.88]	0.89 [0.81–0.94]	0.55 [0.02–0.79]	− 1.21	− 29.0~25.6
Liver Rt lobe 8 cm	4	0.77 [0.60–0.89]	0.82 [0.68–0.91]	0.67 [0.24–0.85]	− 0.41	− 31.7~30.8

Data in parentheses are 95% confidence intervals

*ICC* = intraclass correlation coefficient, *LOA* = limits of agreement

## Discussion

For the phantom study, the intra- and interoperator reproducibility was excellent for both transducers, which indicates high stability in measurement of a homogeneous and still phantom. Based on these results, SWS measurements using the VTQ software are highly reliable for samples under ideal conditions. This suggests that the heterogeneity, movement, size of the target organ, and repeatability of placing the transducer and ROIs might be sources of insufficient reproducibility in humans.

The intraoperator reproducibility in the volunteer study was also excellent for all organs except the pancreas, for which the ICC was fair to good. The greater depth and smaller size of the pancreas in comparison to the other organs measured with the 4C1 transducer might be the reason for lower intraoperator reproducibility.

In contrast, the interoperator ICC was excellent only for the right hepatic lobe at 4 cm depth and kidney. Agreement between the two operators was high for the hepatic lobe at 4 cm depth as the 95% LOA was 25% or less. This result reinforces Barr and colleagues’ comments that the ARFI pulse has a sweet spot at 4–5 cm depth and measurements obtained in this location may have less variability [8]. The decrease in reproducibility with measurement depth in our results is consistent with a previous study measuring focal liver lesions [29].

For the kidney, on the other hand, the 95% LOA was as large as − 43.5~66.9% despite an excellent interoperator ICC. This discrepancy between the 95% LOA and interoperator ICC in the kidney is thought to be due to the inhomogeneity of the renal parenchyma consisting of the cortex and medulla [30]. Reproducibility depends on both the magnitude of measurement error as well as the true heterogeneity in the population from which measurements are made [31]. The wide variability of SWS in the kidney in our study is similar to that measured in previous studies [17, 30, 32, 33].

In our study, the interoperator ICC was as poor as 0.4 or less for cervical lymph node and brachioradialis muscle despite an excellent intraoperator ICC of more than 0.9. The size of the lymph nodes was generally small because the subjects were healthy volunteers. The small size of the lymph node may produce subtle discordance of ROI positioning between the two operators and, hence, differences in SWS measurement and low interoperator reproducibility.

It is reported that the measurement plane and the angle of the transducer relative to the muscle might affect the reproducibility of measurements due to anisotropy of the tissue [34]. In our study, the two operators placed the transducer perpendicular to the body surface along the longitudinal axis of the brachioradialis muscle, so the effect of anisotropy is thought to be small. For the brachioradialis muscle, the small IQR of the SWS estimates is a possible reason for the low interoperator ICC. When analyzing measurement reliability, we suggest that investigators report estimates of within-population IQR and 95% LOA, in addition to the ICC.

There are several studies discussing the reproducibility of SWS measurement in the liver. Our estimate of the intraoperator ICC for the right hepatic lobe at 4 cm depth was similar to that of previous studies using ARFI [12, 35, 36], but our interoperator ICC was worse than that in the results of Woo et al [12] where SWS was measured nine times. Some studies [37, 38] have shown that five SWE measurements could be used instead of ten without a significant effect on diagnostic performance. Other studies [39, 40] have suggested that the optimal minimum number of measurements required was six. Based on the results of our phantom study, where ICCs with ten measurements were higher than those with five measurements, it appears that more measurements may lead to higher stability. Our subjects were healthy volunteers so that the variability is thought to be smaller than that of studies including patients with hepatic disease. As mentioned earlier, as the reliability of a measurement method depends upon the heterogeneity of the population, a direct comparison of the ICC between different populations does not make much sense. In the phantom study, measurement error often occurred for the shallowest ROI (1 cm for the 9L4 transducer and 2 cm for the 4C1 transducer), and thus, those data were abandoned. The measurement error is thought to be due to multiple reflections from the surface of the phantom because the phantoms were wrapped in plastic film to prevent drying [27]. For the volunteer study, the volunteers for whom measurement failure occurred had BMIs of 25.5–29.5, which were four of the top 5 BMIs in the group. The measurement failure is thought to be due to corpulence [41].

Our study has several limitations. We assessed intraoperator reproducibility only within one session of measurement. Ideally, the reproducibility of measurement should also be examined in another session on the same day or the following day (e.g., intraday or interday reproducibility for the same operator), in order to estimate the variation when the same operator repeats the examination. We only performed measurements on normal organ parenchyma. Reproducibility in the examination of tumors, cirrhosis, and other diseases may be different.

We evaluated reproducibility for only one application with one device at one facility. For SWS to be used widely as a reliable biomarker, it would be beneficial to evaluate agreement between different applications, multiple devices, and multiple facilities [42].

In conclusion, our phantom study indicated the high reliability of SWS measurements with pSWE under ideal conditions. For the volunteer study, the intraoperator reproducibility was also generally high, but the interoperator reproducibility from only five measurements was not high enough except for the right hepatic lobe at 4 cm depth. To evaluate interoperator reproducibility, the 95% LOA between operators should be considered in addition to the ICC. It is a great merit of pSWE to be able to evaluate the stiffness quantitatively with ease. To reliably utilize this measurement method clinically, it is essential to have a strong understanding of the degree of reproducibility.

## Electronic supplementary material

ESM 1 (DOCX 209 kb)

## Abbreviations

2D SWE: 2D color-coded shear-wave elastography
ARFI: Acoustic radiation force impulse
BMI: Body mass index
ICC: Intraclass correlation coefficient
IQR: Interquartile range
LOA: Limits of agreement
pSWE: Point shear-wave elastography
QIBA: Quantitative Imaging Biomarkers Alliance
ROI: Region of interest
SWS: Shear-wave speed
TE: Transient elastography
US: Ultrasound
VTQ: Virtual touch quantification
