# Supplementary material for: Intra- and inter-operator reproducibility of US point shear-wave elastography in various organs: evaluation in phantoms and healthy volunteers
Source: Eur Radiol. 2019 May 14;29(11):5999–6008. doi: 10.1007/s00330-019-06195-8 (PMC6795636; doi:10.1007/s00330-019-06195-8)
Supplement: Supplementary file 1 — (DOCX 209 kb) [file 330_2019_6195_MOESM1_ESM.docx]

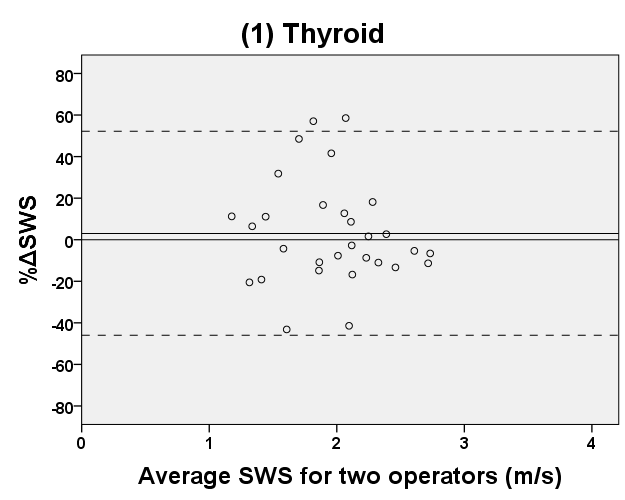

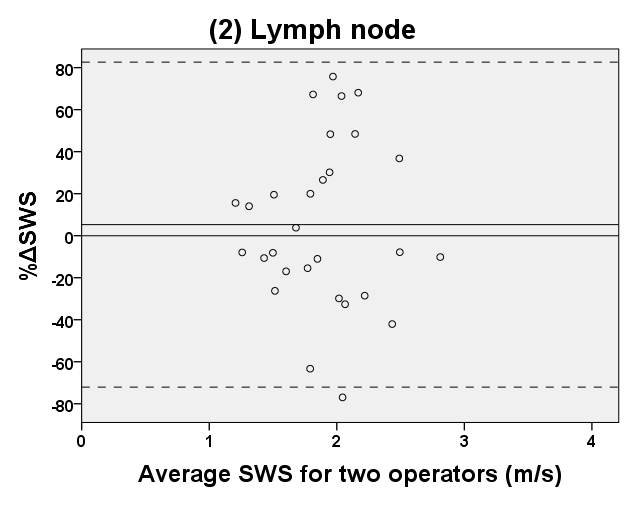


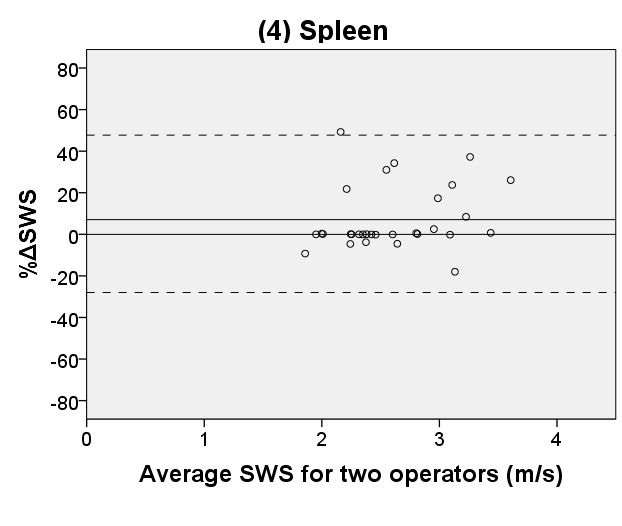

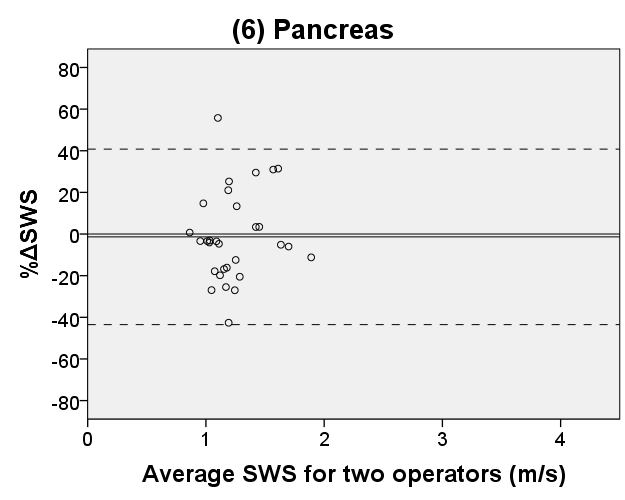

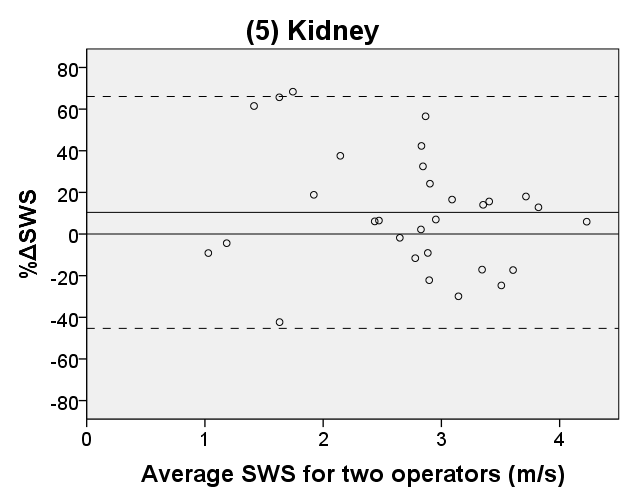

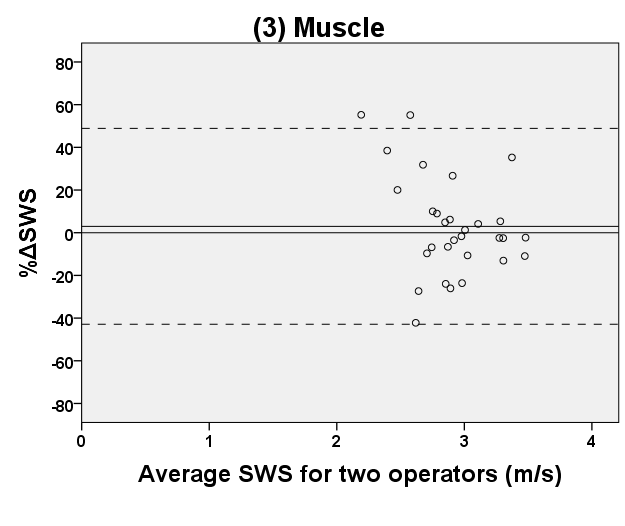


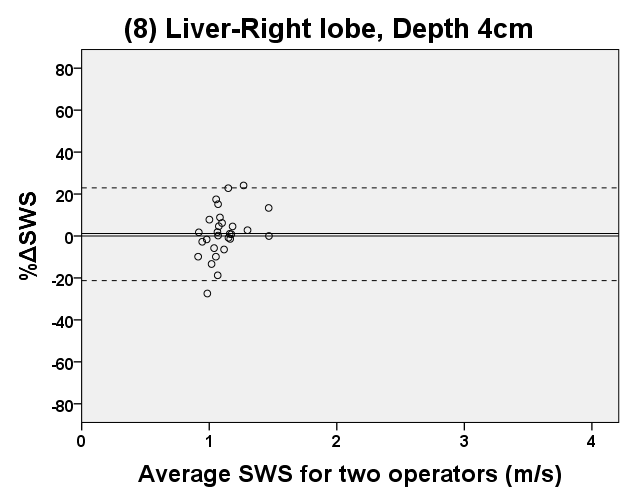

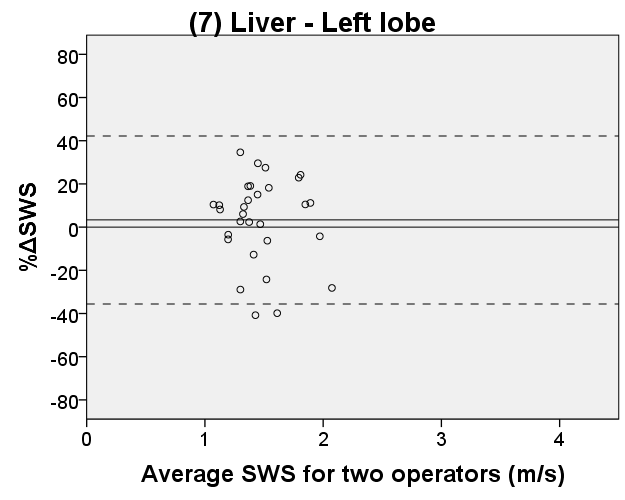

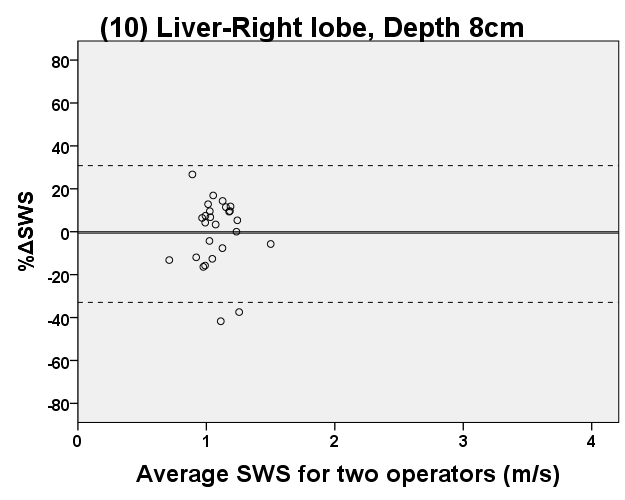

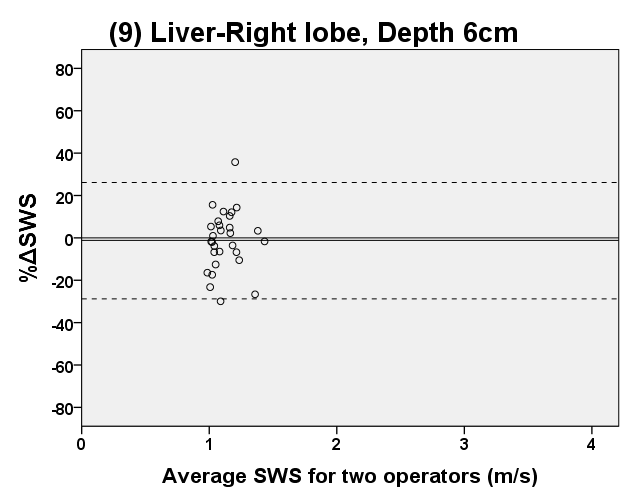


**Supplemental Figure**

Bland-Altman plots for inter-operator reproducibility of shear wave speed (SWS) measurement in the volunteer study. The X-axis corresponds to the mean SWS for two operators and the Y axis is %ΔSWS. Solid line = mean bias, Dashed line = 95% limits of agreement (1.96 x standard deviation)

(1) Thyroid (2) Cervical lymph node (3) Brachioradialis muscle (4) Spleen (5) Left kidney (6) Pancreas (7) Left hepatic lobe (8) Right hepatic lobe, at 4-cm depth (9) Right hepatic lobe, at 6-cm depth (10) Right hepatic lobe, at 8-cm depth
